# Focal Choroidal Excavation

**DOI:** 10.4274/tjo.24445

**Published:** 2016-12-01

**Authors:** Zafer Cebeci, Şerife Bayraktar, Merih Oray, Nur Kır

**Affiliations:** 1 İstanbul University İstanbul Faculty of Medicine, Department of Ophthalmology, İstanbul, Turkey

**Keywords:** optical coherence tomography, central serous chorioretinopathy, choroidal neovascularization

## Abstract

Focal choroidal excavation is a choroidal pit that can be detected by optical coherence tomography. Central serous chorioretinopathy, choroidal neovascularization and polypoidal choroidal vasculopathy are pathologies associated with focal choroidal excavation. In this article, we present the follow-up and treatment outcomes of three eyes of two patients with focal choroidal excavation.

## INTRODUCTION

Focal choroidal excavation is local idiopathic cupping of the choroid which is usually unilateral and not associated with any accompanying systemic disease.^1^ In 2006, Jampol et al.^2^ first identified the lesion in an asymptomatic patient using optic coherence tomography (OCT). Margolis et al.^3^ later used the term focal choroidal excavation for the areas of choroidal pitting observed near the macula on spectral domain (SD)-OCT in patients without posterior staphyloma or scleral ectasia. The condition causes symptoms like decreased vision and metamorphopsia, but its etiology is not fully understood. Studies have documented that focal choroidal excavation may be accompanied by choroidal vascular disorders including central serous chorioretinopathy (CSCR), choroidal neovascularization (CNV) and polypoidal choroidal vasculopathy (PCV), which are responsible for the visual symptoms.^4,5,6,7,8^

In this report we present the treatment and follow-up results of three eyes of two patients with the rare condition of focal choroidal excavation.

## CASE REPORTS

### Case 1

**Figure 1:**
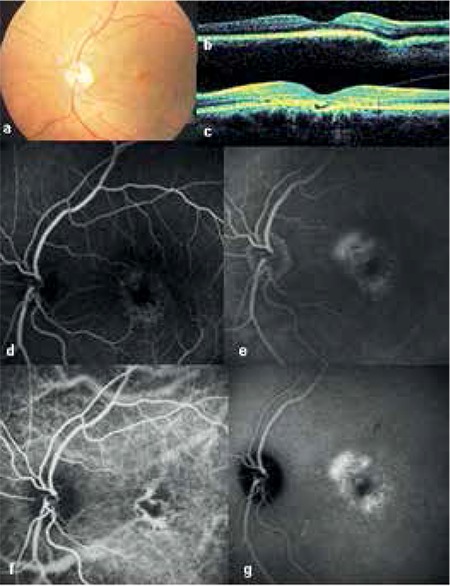
Case 1 before treatment: left eye fundus photography showing macular pigment epithelium changes (a), right eye optic coherence tomography showing focal choroidal excavation (b), left eye optic coherence tomography showing subfoveal choroidal excavation and subretinal fluid (c), left eye fundus fluorescein angiography showing early stage hyperfluorescence as a window defect (d), left eye fundus fluorescein angiography showing intensified hyperfluorescence in the late phase (e), left eye indocyanine green angiography showing choroidal vessel dilation in the early phase (f), left eye indocyanine green angiography showing late phase hyperfluorescence due to leakage from choroidal vessels (g)

A 50-year-old female patient presented to the Ophthalmology Department of the İstanbul University İstanbul Faculty of Medicine with an approximately 2-year history of gradual vision loss in her left eye. Despite progressively decreasing vision in her left eye over the course of 2 years, she had not previously consulted any doctor about the problem. There was nothing extraordinary in the patient’s medical or family history. Her vision was 1.0 (decimal) in the right eye and 0.05 in the left eye. Anterior segment examination was normal and intraocular pressure was 15 mmHg in the right eye and 16 mmHg in the left eye. Pigment epithelium changes were observed in both the right and left macula on fundoscopy (Figure 1a). OCT examination revealed extrafoveal inferonasal choroidal excavation in the right eye (Figure 1b), while in the left eye subfoveal focal choroidal excavation was observed, as well as separation of the retinal pigment epithelium (RPE) photoreceptor layer and subretinal fluid in the same area (Figure 1c). Central foveal thickness was 245 µm in the left eye. Fundus fluorescein angiography (FFA) and indocyanine green angiography (ICGA) revealed hyperfluorescence consistent with pigment epithelium window defect in the macula and temporal quadrant of the right eye, and hyperfluorescence starting in the early phase and increasing in the late phases in the left macula (Figure 1d, 1e, 1f, 1g). The patient was diagnosed with chronic CSCR and her left eye was treated with low-fluence photodynamic therapy (PDT) (25 j/cm^2^, 300 mW/cm^2^). The spot size was adjusted targeting the area of choroidal vascular hyperpermeability observed in the ICGA mid-phase from which the subretinal fluid originated.

**Figure 2:**
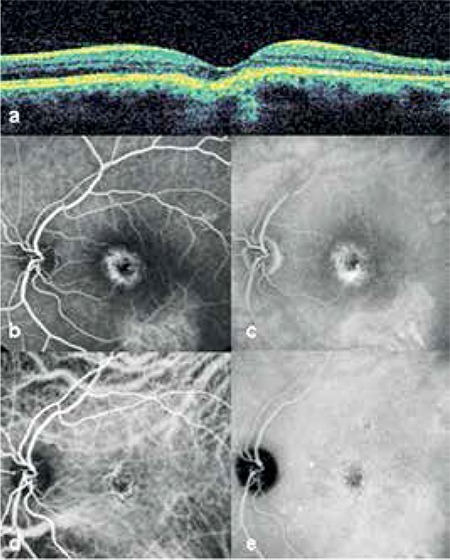
Case 1 at 18 months after photodynamic therapy: focal choroidal excavation and subretinal fluid do not appear on left eye optic coherence tomography (a), left eye fundus fluorescein angiography, early phase (b), left eye fundus fluorescein angiography showing late phase hyperfluorescence due to a window defect (c) left eye indocyanine green angiography showing resolution of choroidal vessel dilation in the early phase (d), late phase hyperfluorescence is no longer apparent on left eye indocyanine green angiography

At 1 month after PDT, the subretinal fluid had resolved and visual acuity was 0.3. After 18 months of follow-up, no changes were observed in the lesions in the right eye, visual acuity in the left eye as maintained and there was no recurrence of subretinal fluid. OCT at the final examination showed continuity of the RPE and photoreceptor layer in the area of focal choroidal excavation, and no subretinal fluid was observed (Figure 2a). On FFA, the hyperfluorescence due to RPE window defect was not evident (Figure 2b, 2c) and there was no active leakage apparent on ICGA (Figure 2d, 2e).

### Case 2

**Figure 3:**
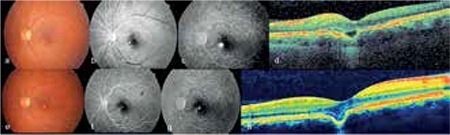
Case 2 pre- and post-treatment appearance: left eye fundus photography showing macular pigment epithelium changes (a), left eye fundus fluorescein angiography, early phase (b), left eye fundus fluorescein angiography showing hyperfluorescence intensifying in the late phase (c), left eye optic coherence tomography showing focal choroidal excavation and an overlying hyporeflective area (d), left eye fundus photograph at 2 years post-treatment showing pigment epithelium changes at the macula (e), left eye fundus fluorescein angiography, early phase at 2 years post-treatment (f), left eye fundus fluorescein angiography at 2 years post-treatment showing hyperfluorescence slightly intensified in the late phase (g), left eye optic coherence tomography at 2 years post-treatment showing conforming excavation and the absence of a hyporeflective area (h)

A 28-year-old female patient presented with metamorphopsia in her left eye starting 2 days earlier. There was nothing of note in her medical or family history, and her visual acuity was 1.0 in the right eye and 0.8 with -5.0 D refraction in the left eye. Anterior segment examination was normal. Fundus examination was normal in the right eye, but macular pigmentary alterations were observed in the left eye (Figure 3a). On FFA there was hyperfluorescence beginning in the early phases and increasing in the late phase, which was more suggestive of choroidal neovascular membrane (CNVM) than CSCR (Figure 3b, 3c). Although it is recommended for a definitive diagnosis, ICGA was not done. Despite smooth foveal contours in the left eye on OCT, an area of subfoveal focal choroidal excavation and overlying hyporeflective subretinal fluid were detected (Figure 3d). The lesion in the patient’s left eye was accepted as CNVM and an intravitreal bevacizumab injection was administered. At follow-up 1 month later, the patient’s symptoms had improved, vision in her left eye improved to 0.9 and the hyporeflective area evident on OCT had decreased in size. At 2-year follow-up, visual acuity in the left eye was 0.8 and persistent RPE changes were observed on fundoscopy (Figure 3e). Hyperfluorescence which increased slightly in the late phases was observed on FFA of the left eye (Figure 3f, 3g). On OCT, the focal choroidal excavation remained unchanged, the overlying hyporeflective area had resolved and the photoreceptor layer appeared continuous (Figure 3h).

## DISCUSSION

Focal choroidal excavation is a choroidal defect believed to be a congenital condition, though its etiology and pathogenesis are not yet fully understood, and is detectable on SD-OCT.^1^ This excavation has been termed ‘nonconforming’ if photoreceptors are detached from the RPE, or ‘conforming’ when the RPE follows the contours of the photoreceptor layer.^3^ The nonconforming type exhibits a hyporeflective space on SD-OCT which does not appear in the conforming type.

Focal choroidal excavation is generally a stable, unchanging lesion.^1^ Our patient with bilateral involvement also had extrafoveal excavation in the fellow eye, but visual acuity was not affected and no complications resulted.

CSCR, CNV and PCV are all pathologies which may accompany focal choroidal excavation.^4,5,6,7,8^ It has not been determined whether CSCR leads to focal choroidal excavation or whether CSCR occurs as a complication of excavation. One of the proposed mechanisms is that excavation is mainly responsible for the pathology, causing atrophy of the overlying RPE and subsequent pump dysfunction, and CSCR occurs as a complication.^7^ It has also been proposed that CNV and PCV are both the result of choroidal ischemia in areas of anatomic anomalies.^1^

In a report from Margolis et al.^3^ including 12 patients, CSCR was detected in 1 patient who later developed CNV during follow-up. Suzuki et al.^7^ evaluated 7 eyes of 6 patients with CSCR and focal choroidal excavation. Although the subretinal fluid resolved in all cases, 3 patients later progressed to nonconforming excavation, which exhibits the same hyporeflectivity on OCT as subretinal fluid. The authors attributed this to persistent subretinal fluid around the lesion. In their series of 41 eyes, Lee et al.^1^ detected CSCR in 10 eyes, CNV in 9 eyes and PCV in 1 eye; 2 eyes with CSCR were treated with low-fluence PDT. Despite resolution of the subretinal fluid in these patients, they continued to exhibit separation of the RPE and photoreceptor layer (nonconforming type). The nonconforming type was shown to be significantly associated with visual symptoms and CSCR.^1^ This was also true in our two patients, who exhibited nonconforming excavation and experienced visual symptoms. They both reverted to the conforming type, after PDT in the first case and after intravitreal bevacizumab injection in the second case. The resolution of the hyporeflective area evident on OCT in both patients may be related to the decreased choroidal permeability following PDT in the first patient and resolution of the active focal exudation in the retina after intravitreal bevacizumab injection in the second patient. The fact that patients transition between types supports the idea that hyporeflective areas on OCT in the nonconforming type may be due to subretinal fluid. Neither of our patients fully regained their vision after treatment, which may be attributable to the presence of chronic CSCR and subsequent RPE dysfunction.

## CONCLUSION

In one eye of our first case, focal choroidal excavation remained static over the course of follow-up and did not require treatment. The same patient’s other eye was treated with low-fluence PDT and the affected eye of our second case was treated with intravitreal bevacizumab; both eyes showed regression to conforming excavation after treatment. Studies with larger patient numbers and longer follow-up times are needed to better understand the etiology, course and treatment options of focal choroidal excavation.

### Ethics

Informed Consent: It was taken.

Peer-review: Externally and internally peer-reviewed.

## References

[1] Lee CS, Woo SJ, Kim YK, Hwang DJ, Kang HM, Kim H, Lee SC (2014). Clinical and spectral-domain optical coherence tomography findings in patients with focal choroidal excavation. Ophthalmology..

[2] Jampol LM, Shankle J, Schroeder R, Tornambe P, Spaide RF, Hee MR (2006). Diagnostic and therapeutic challenges. Retina..

[3] Margolis R, Mukkamala SK, Jampol LM, Spaide RF, Ober MD, Sorenson JA, Gentile RC, Miller JA, Sherman J, Freund KB (2011). The expanded spectrum of focal choroidal excavation. Arch Ophthalmol..

[4] Obata R, Takahashi H, Ueta T, Yuda K, Kure K, Yanagi Y (2013). Tomographic and angiographic characteristics of eyes with macular focal choroidal excavation. Retina..

[5] Kobayashi W, Abe T, Tamai H, Nakazawa T (2012). Choroidal excavation with polypoidal choroidal vasculopathy: a case report. Clin Ophthalmol..

[6] Xu H, Zeng F, Shi D, Sun X, Chen X, Bai Y (2014). Focal choroidal excavation complicated by choroidal neovascularization. Ophthalmology..

[7] Suzuki M, Gomi F, Hara C, Sawa M, Nishida K (2014). Characteristics of central serous chorioretinopathy complicated by focal choroidal excavation. Retina..

[8] Say EA, Jani PD, Appenzeller MF, Houghton OM (2013). Focal choroidal excavation associated with polypoidal choroidal vasculopathy. Ophthalmic Surg Lasers Imaging Retina..

